# Effect of heme oxygenase-1 polymorphisms on lung function and gene expression

**DOI:** 10.1186/1471-2350-12-117

**Published:** 2011-09-08

**Authors:** Goh Tanaka, Farzian Aminuddin, Loubna Akhabir, Jian-Qing He, Karey Shumansky, John E Connett, Nicholas R Anthonisen, Raja T Abboud, Peter D Paré, Andrew J Sandford

**Affiliations:** 1 UBC James Hogg Research Center, Providence Heart + Lung Institute, St. Paul's Hospital, Vancouver, B.C., Canada; 2 Division of Biostatistics, School of Public Health, University of Minnesota, Minneapolis, MN, USA; 3 Faculty of Medicine, University of Manitoba, Winnipeg, MB, Canada; 4 Division of Respiratory Medicine, Department of Medicine, Vancouver General Hospital, UBC, Vancouver, B.C., Canada

**Keywords:** Heme oxygenase, polymorphism, chronic obstructive pulmonary disease

## Abstract

**Background:**

Oxidative stress induced by smoking is considered to be important in the pathogenesis of Chronic Obstructive Pulmonary Disease (COPD). Heme oxygenase-1 (HMOX1) is an essential enzyme in heme catabolism that is induced by oxidative stress and may play a protective role as an antioxidant in the lung. We determined whether *HMOX1 *polymorphisms were associated with lung function in COPD patients and whether the variants had functional effects.

**Methods:**

We genotyped five single nucleotide polymorphisms (SNPs) in the *HMOX1 *gene in Caucasians who had the fastest (n = 278) and the slowest (n = 304) decline of FEV_1 _% predicted, selected from smokers in the NHLBI Lung Health Study. These SNPs were also studied in Caucasians with the lowest (n = 535) or the highest (n = 533) baseline lung function. Reporter genes were constructed containing three *HMOX1 *promoter polymorphisms and the effect of these polymorphisms on H_2_O_2 _and hemin-stimulated gene expression was determined. The effect of the *HMOX1 *rs2071749 SNP on gene expression in alveolar macrophages was investigated.

**Results:**

We found a nominal association (p = 0.015) between one intronic *HMOX1 *SNP (rs2071749) and lung function decline but this did not survive correction for multiple comparisons. This SNP was in perfect linkage disequilibrium with rs3761439, located in the promoter of *HMOX1*. We tested rs3761439 and two other putatively functional polymorphisms (rs2071746 and the (GT)_n _polymorphism) in reporter gene assays but no significant effects on gene expression were found. There was also no effect of rs2071749 on *HMOX1 *gene expression in alveolar macrophages.

**Conclusions:**

We found no association of the five *HMOX1 *tag SNPs with lung function decline and no evidence that the three promoter polymorphisms affected the regulation of the *HMOX1 *gene.

## Background

Oxidative stress induced by smoking is considered to play a role in the pathogenesis of Chronic Obstructive Pulmonary Disease (COPD). Oxidant compounds in cigarette smoke cause an excess of oxidants in the lung, and lead to direct cell injury, lung extracellular matrix damage, inactivation of antiproteinases, and induction of proinflammatory mediators [1].

Heme oxygenase-1 (HMOX1) is an essential enzyme in heme catabolism and is induced by oxidative stress. HMOX1 catalyzes the conversion of heme to biliverdin, carbon monoxide and iron. Subsequent to the reaction, biliverdin is converted to bilirubin by biliverdin reductase. Biliverdin and bilirubin have been shown to act as scavengers of reactive oxygen species, and carbon monoxide has also been shown to possess anti-inflammatory effects (reviewed in [2]). In both *in vitro *and *in vivo *studies, murine cells lacking Hmox1 have been reported to be susceptible to oxidative injury [3], and exogenous delivery of Hmox1 by gene transfer in the rat lung was shown to provide protection against hyperoxia-induced injury [4]. The results of these studies suggest that HMOX1 has a protective role as an antioxidant in the lung.

Genetic risk factors are thought to contribute to the impact of smoking by modulating the severity of the smoke-induced injury and thus the severity of chronic airflow obstruction [5]. Antioxidant genes have been considered to be plausible candidate genes for this susceptibility [6]. In this study, we investigated whether variations of the *HMOX1 *gene were associated with the level of lung function in smokers who had mild to moderate airway obstruction. To identify potential causal variants in the *HMOX1 *gene, we analyzed a set of highly informative single nucleotide polymorphism (SNP) markers which covered the gene including the promoter region.

## Methods

### Genetic association analysis in the Lung Health Study

#### Study participants

The National Heart, Lung, and Blood Institute Lung Health Study (LHS) is a multicenter clinical study with 5,887 participants who had spirometric evidence of mild-moderate lung function impairment [6,7]. The lung function values for each participant were compared to the predicted normal values of Crapo *et al. *[8]. Lung function impairment was defined by a forced expiratory volume in one second (FEV_1_) to forced vital capacity (FVC) ratio of ≥ 0.70 and FEV_1 _55%-90% of predicted. Participants of European descent were selected from the cohort, and two nested case control studies were designed based on the rate of lung function decline and the lung function at the start of the LHS. First, individuals who had the most and least rapid rate of decline of lung function were selected from those who continued to smoke for the duration of the 5 years follow up. Those whose FEV_1_% predicted decreased by ≥ 3.0%/year during the 5 year period (fast decline group, n = 278) were compared with those whose FEV_1_% predicted increased ≥ 0.4%/year (non-decline group, n = 304). Second, subjects who had the highest post-bronchodilator FEV_1_% predicted (≥ 88.9%; high function group, n = 533) and subjects who had lowest post-bronchodilator FEV_1_% predicted (≤ 67.0%; low function group, n = 535) at the start of the LHS were compared. Since 139 subjects included in the fast or non-decline groups had baseline lung function within these criteria, they were also included in the baseline function study. Informed consent was obtained from each individual, and the present study protocol was approved by the Providence Health Care Research Ethics Board.

#### TagSNP selection and genotyping

**Figure 1:**
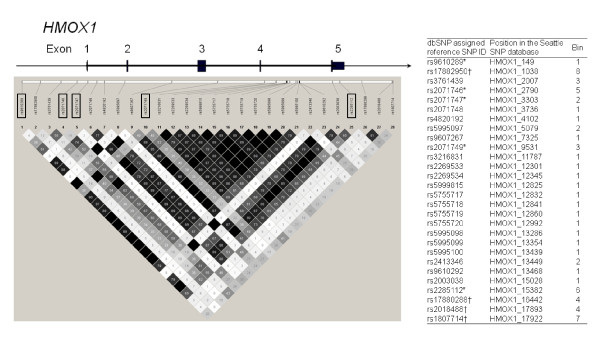
**Single-nucleotide polymorphisms (SNPs) in the region of the heme oxygenase 1 (*HMOX1*) gene whose allele frequency was > 10% in European-American Coriell samples**. Shading represents the strength of pair-wise linkage disequilibrium (LD) with a black-to-white gradient reflecting higher to lower r^2 ^values. Bin: a group of SNPs where the alleles are highly associated. *: tag SNPs; †: SNPs which were excluded from the study.

Information from the SeattleSNPs website http://pga.gs.washington.edu was used for the selection of the *HMOX1 *SNPs. There were 28 SNPs whose minor allele frequency was more than 10% in the *HMOX1 *gene sequence (GenBank accession# AY460337). Pairwise linkage disequilibrium (LD) statistics for the SNPs were visualized by the Haploview 4.1 software [9] and are shown in Figure 1. TagSNPs were chosen from these SNPs using the ldSelect program (version 1.0) [10]. An LD threshold of r^2 ^> 0.8 was set in the program. Assays to genotype rs17882950, rs17880288, rs2018488 and rs1807714 could not be established and therefore these SNPs were excluded from the study. In total, five SNPs were genotyped using TaqMan assays (Applied Biosystems, Carlsbad, CA). These SNPs tagged 86% (24/28) of the SNPs present in this region in the PGA European panel. The only coding SNP among these tags was rs2071747 that resulted in a change of amino acid from aspartate to histidine at position 7 of the polypeptide. As expected there was no strong LD between the five tag SNPs although rs2071746 showed moderate LD with rs9610289 and rs2071749 (r^2 ^= 0.60 and 0.65, respectively). The LD between all other pairs of SNPs was r^2 ^< 0.40.

### Detection of potential transcription factor binding sites in the promoter sequence

The online software tool, MatInspector [11] was used to identify consensus sequences for transcription factor binding sites. This tool is available at http://www.genomatix.de.

### Transient-Transfection Assay

#### Construction of reporter gene vectors

rs2071746 and the GT repeat polymorphism, which have been reported as functional variants in previous studies [12,13], are located between rs3761439 and the transcription start site. We conducted reporter gene assays to elucidate the possible effect of these 3 polymorphisms on the promoter activity. Since rs3761439 was in complete LD (D' = 1) with rs2071746, we excluded the rs3761439A - rs2071746T haplotype which was expected not to exist in our subjects. We selected 23, 30, and 37 GT repeats as representative alleles of small (S) (< 27 repeats), medium (M) (27 - 32 repeats), and large (L) (≥ 33 repeats) classes, respectively because these were the most common alleles of those classes in our study population.

A 1.3 kb fragment containing nucleotides -1,301 to +22 from the transcription start site of the *HMOX1 *gene, including the rs3761439 and rs2071746 SNPs and the (GT)_n _repeat polymorphism, was amplified by polymerase chain reaction (PCR) using the forward primer 5'-ATT CGC TAG CCA TCC CAG GCT CAA GTG AAC-3' and the reverse primer 5'-CTG AGG ACG CTC GAG AGG A-3'. PCR was performed using *PfuUltra *High-Fidelity DNA Polymerase (Stratagene, La Jolla, CA) as described by the manufacturers. The PCR products were then digested with *Nhe*I and *Xho*I, and each fragment was cloned into the pGL3-Basic vector (Promega, Madison, WI) between these restriction sites.

**Figure 2:**
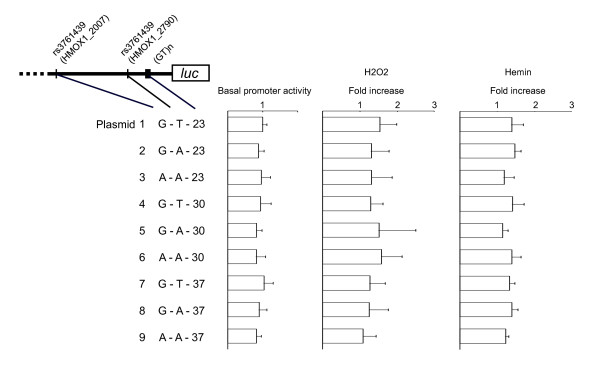
***HMOX1 *gene promoter analysis of 9 different constructs**. Each test construct was co-transfected into A549 cells with Renilla luciferase vector as an internal control. The basal promoter activity of each plasmid is indicated as luciferase activity relative to that of construct 1. The relative inducibility by H_2_O_2 _(300 μM) or Hemin (100 μM) is shown for each construct. Data are presented as means + SD from three to four independent experiments. There was no significant difference in induction of reporter gene expression among these constructs.

Site-specific mutation for rs2071746 was performed to make plasmids 2, 4, and 8 (see Figure 2) following the protocol of the QuikChange II site-directed mutagenesis kit (Stratagene, La Jolla, CA). Primers for the mutagenesis reactions were: 5'-GCC CAC CAG GCT ATT GCT CTG AGC AGC G-3' and 5'-CGC TGC TCA GAG CAA TAG CCT GGT GGG C-3' for plasmids 2 and 8; 5'-GCC CAC CAG GCT TTT GCT CTG AGC AGC G-3' and 5'-CGC TGC TCA GAG CAA AAG CCT GGT GGG C-3' for plasmid 4.

To construct plasmids 3 and 9, sequences that had 23 and 37 GT repeats were digested with *Pst*I and *Xho*I, and each fragment was cloned into plasmid 6 (which originally had 30 GT repeats between these restriction sites).

The sequence fidelity of each construct was confirmed by DNA sequencing. Quantification of plasmid DNA concentration was performed using Quant-iT PicoGreen dsDNA reagent and kits (Invitrogen, Carlsbad, CA).

#### Transfection and luciferase assay

A549 cells (American Type Culture Collection number CRL-1848) were cultured in Dulbecco's modified Eagle medium supplemented with 10% fetal bovine serum. For luciferase assay 5 × 10^4 ^cells were plated on 24-well plates, and were cultured overnight. The plasmids were transfected into A549 cells using Fugene 6 nonliposomal reagent, according to the manufacturer's protocol (Roche Diagnostics, Lewes, UK). After 24 h, the cells were treated with medium with or without stimuli for another 18 h. H_2_O_2 _(300 μM) and hemin (100 μM) were used as stimuli. Hemin was dissolved in medium with 0.5% DMSO, and the control for the hemin was treated with the same medium without hemin. The cells were then harvested and assayed following the Dual-Luciferase reporter assay system protocol (Promega, Madison, WI). Luciferase activity was corrected for transfection efficiency using a co-transfected renilla-luciferase vector with the experimental plasmid at a ratio of 1:10. Three or four independent experiments were carried out, and each assay was performed in triplicate.

### *HMOX1 *expression in alveolar macrophages

#### Samples

Genomic DNA and total RNA were isolated from alveolar macrophages of 58 individuals who underwent lung resection for a small (< 3 cm), Stage I or II, peripheral tumor at Vancouver General Hospital between July 2001 and November 2004, as described previously [14]. This study was approved by the University of British Columbia/Providence Health Care and Vancouver Hospital and Health Sciences Research Ethics Boards and all subjects provided written informed consent.

The resected lung or lobe was obtained immediately post-operatively and bronchoalveolar lavage was performed. Details of the lavage, macrophage separation and subsequent culture are as described previously [14]. For each sample, we assessed the following conditions: fresh alveolar macrophages, non-stimulated macrophages after 24 hours of culture, and LPS-stimulated macrophages after 24 hours of culture.

#### Genotyping

Genomic DNA from the alveolar macrophages was genotyped for the rs2071749 SNP by TaqMan assay (Applied Biosystems).

#### Quantitative PCR

cDNA samples were prepared from alveolar macrophages and were used to determine the gene expression of *HMOX1*. The reference gene used was *GNB2L1*, a guanine nucleotide-binding protein as this was previously shown to be stably expressed in alveolar macrophages [15]. Gene expression assays for *HMOX1 *(Hs00157965_m1) and *GNB2L1 *(Hs00272002_m1) were from Applied Biosystems.

### Statistical Analysis

We examined whether genotype frequencies were compatible with Hardy-Weinberg equilibrium using the genetics package for R http://www.r-project.org. Fisher's exact test was performed for the 3-by-2 tables of the codominant model using R. Multivariate logistic regression was used to adjust for age, sex, pack-years of smoking, and the recruiting research center. Haplotype frequencies were estimated with an EM algorithm using the R haplo.stats package. Haplotype association was performed using hapassoc [16], a contributed R package.

For the transient transfection assay data, the Kruskal-Wallis test was used to compare fold increase of luciferase activity by H_2_O_2 _or hemin. For testing the 9 plasmids, the p value was confirmed by examining the distribution of Kruskal-Wallis statistics using randomly-generated data through computer simulation (10,000 permutations). For assessment of statistical significance of each SNP in the assay, the Wilcoxon rank-sum test was used. A p value < 0.05 was considered significant.

For the analysis of gene expression in alveolar macrophages, a relative expression ratio was calculated from the real-time PCR efficiencies and the cycle threshold (C_T_) values for the samples, as described previously [17]. All three genotypes (GG n = 14; AG n = 32; AA n = 12) were examined to compare relative expression of *HMOX1*. The *JMP 5.1 *statistical software package (SAS Institute Inc., Cary, NC, USA) was used for assessment of statistical significance of *HMOX1 *expression among the three genotypes. A p value < 0.05 was considered significant.

## Results

### Characteristics of the LHS study participants

The characteristics of the LHS participants are shown in Table 1. Several potentially confounding factors were significantly different between the fast and non decline groups and between the low and high baseline groups, as reported previously [18].

**Table 1:** Characteristics of participants in the rate of lung function decline sub-study and the baseline lung function sub-study

	Rate of decline study			Baseline study		
		
	Fast decline (n = 278)	Non decline (n = 304)	P value	Low function (n = 535)	High function (n = 533)	P value
Female/Male	115/163	101/203	0.04	205/330	180/353	0.12
Age (years)	49.5 ± 6.7	47.6 ± 7.0	0.0005	50.7 ± 6.9	46.2 ± 6.9	< 0.0001
Smoking (pack-years)	43.0 ± 18.3	38.3 ± 17.4	0.003	45.3 ± 18.5	35.4 ± 18.5	< 0.0001
ΔFEV_1_, % predicted/yr*	-3.44 ± 1.17	0.68 ± 1.05	< 0.0001	-0.78 ± 1.85	-0.75 ± 1.39	0.73
Baseline FEV_1_, % predicted†	74.8 ± 8.3	80.0 ± 8.7	< 0.0001	62.6 ± 2.3	91.8 ± 2.3	< 0.0001

Data are presented as means ± SD. Lung function was measured after administration of bronchodilator.

* Change in lung function per year over a 5 year period

† Lung function at the start of the Lung Health Study

### Single SNP analysis in the LHS

We analyzed each of the selected SNPs in the rate of lung function decline sub-study and the baseline lung function sub-study (Table 2). A multivariate logistic regression model was used to adjust for confounding factors including age, gender, pack-years of smoking, and research center in the analyses. All the SNPs were in Hardy-Weinberg equilibrium. We found a nominally significant difference in genotype frequencies of rs2071749 between the fast and non decline groups (Table 2). However, this association was not significant after correction for multiple comparison (p = 0.075). In addition, the rs2071749 SNP was not associated with rate of decline of lung function expressed as a continuous variable (change in FEV_1_% predicted) in the fast decline group (p = 0.41).

**Table 2:** Single nucleotide polymorphisms (SNPs) in the *HMOX1 *region genotyped in this study

		MAF in the rate of decline study			MAF in the baseline study		
					
SNP	Alleles (major/minor)	Fast decline	Non decline	P value*	Low function	High function	P value*
rs9610289	G/A	0.301	0.311	0.689	0.342	0.342	0.894
rs2071746	A/T	0.405	0.434	0.223	0.429	0.436	0.931
rs2071747	G/C	0.058	0.065	0.163	0.050	0.046	0.656
rs2071749	G/A	0.495	0.452	**0.015**	0.475	0.458	0.184
rs2285112	A/G	0.356	0.370	0.572	0.397	0.395	0.933

MAF = minor allele frequency

* P values in the table are for the codominant model and are adjusted by multivariate logistic regression for age, sex, pack-years of smoking, and research center.

The LD in the LHS dataset between the SNPs and the GT repeat polymorphism previously reported to be associated with emphysema [13] is shown in Table 3. The repeat polymorphism was stratified into two "alleles" based on repeat length i.e. the L allele as previously described [13] vs. all other alleles. In this analysis, only the rs2071747 SNP was in high LD with the repeat polymorphism (Table 3).

**Table 3:** Linkage disequilibria between the *HMOX1 *single nucleotide polymorphisms (SNPs) and the GT repeat polymorphism previously reported to be associated with emphysema [13]

SNP	Linkage disequilibrium statistic		
	**D'**	**r^2^**	**P value**

rs9610289	0.916	0.0285	< 0.001
rs2071746	0.932	0.0920	< 0.001
rs2071747	0.985	0.8309	< 0.001
rs2071749	0.891	0.0547	< 0.001
rs2285112	0.839	0.0308	< 0.001

### Haplotype analysis in the LHS

We analyzed the distribution of haplotypes of the five *HMOX1 *SNPs (Table 4). As in the single SNP analyses, we adjusted for potential risk and confounding factors. There was no significant association between the *HMOX1 *haplotypes and lung function in the two studies.

**Table 4:** Haplotype analysis of the *HMOX1 *gene

	Rate of decline study			Baseline study		
	
Haplotype^†^	Fast decline	Non decline	P value*	Low function	High function	P value*
ATGGG	0.299	0.309	0.71	0.326	0.342	0.47
GAGAA	0.492	0.450		0.474	0.455	
GAGGA	0.095	0.111		0.084	0.100	
GTCGA	0.057	0.064		0.048	0.048	
GTGGG	0.046	0.056		0.051	0.042	

Haplotype frequencies were estimated with an EM algorithm implemented in haplo.stats.

† Haplotypes formed from polymorphisms in the following order: rs9610289, rs2071746, rs2071747, rs2071749, rs2285112.

* P values are adjusted by multivariate logistic regression for age, sex, pack-years of smoking, and research center. P values are derived from a global test of the difference in distribution of the five haplotypes between the two phenotypic groups.

### Promoter sequence analysis

The tagSNP, rs2071749, which is located in intron 3 in the *HMOX1 *gene, was in perfect LD (r^2 ^= 1) with rs3761439, which is in the promoter region. We analyzed the promoter region using tools which detect consensus sequences for transcription factor binding sites. We found that rs3761439 changed the consensus sequence of an NF-κB binding site [GGG(A/G)NN(T/C)(T/C)CC]. The matrix similarity of the rs3761439G sequence (Ggggtctccc) for NF-κB was 0.888, which was higher than the optimized matrix similarity thresholds, while that of the rs3761439A sequence (Agggtctccc) was lower than 0.60, which was the lowest threshold of the software.

### Transient transfection assay

The results of the transient transfection assay are shown in Figure 2. There were no significant differences in the promoter activity between the constructs under basal conditions. We found a significant increase of luciferase activity following stimulation by H_2_O_2 _and hemin, however, we did not detect any significant difference in the relative inducibility among the constructs (p = 0.91 for H_2_O_2 _stimulation, p = 0.42 for hemin stimulation). There was also no significant difference in the inducibility in single locus assessment of each polymorphism e.g. for rs3761439 comparing plasmids 2, 5, and 8 (allele G) vs. plasmids 3, 6 and 9 (allele A).

### *HMOX1 *expression in alveolar macrophages

The genotype distribution of the rs2071749 SNP fulfilled the Hardy Weinberg equilibrium (GG (0.25), AG (0.56), AA (0.21); p = 0.71).

In the fresh alveolar macrophages, the AG genotype of rs2071749 was associated with 1.28 fold higher *HMOX1 *gene expression while the AA genotype was associated with a 1.06 fold higher gene expression in comparison to the GG reference group, but these differences were not significant. In the non-stimulated alveolar macrophages after 24 hours in culture, the AG and AA genotypes were associated with higher *HMOX1 *expression by 1.10 and 1.24 fold, respectively, when compared to the GG genotype but these differences were not significant. Similarly, in the LPS-stimulated alveolar macrophages both the AG and AA genotypes showed higher expression of 1.19 and 1.12 fold, respectively, with respect to the GG reference group. However, these differences were not significant. There was also no significant difference in the change in gene expression i.e. comparing LPS-stimulated HMOX1 expression minus non-stimulated expression.

## Discussion

We found a borderline significant association between one tagSNP, rs2071749, and lung function decline in smokers who had mild to moderate airway obstruction. This intronic tagSNP was in perfect LD (r^2 ^= 1) with rs3761439, which is located in the promoter region of the *HMOX1 *gene. We tested rs3761439 and two other putatively functional polymorphisms (rs2071746 and the (GT)_n _polymorphism) in reporter gene assays but no effects on gene expression were found.

The (GT)_n _polymorphism in the *HMOX1 *gene was investigated in a Japanese study population [13] and the L class of alleles (≥ 33 repeats) was significantly associated with pulmonary emphysema in smokers [13]. The (GT)_n _polymorphism was also associated with level of lung function in early-onset COPD families and with COPD [19] but not with measures of functional impairment [20]. In the LHS, no association was found between (GT)_n _repeat classes and the rate of decline of lung function [6]. In contrast, Guenegou *et al. *reported an association between lung function decline and the (GT)_n _polymorphism in the European Community Respiratory Health Survey (ECRHS) [21]. Similar results were reported in Japanese [22] and Dutch populations [23].

Lung function traits were investigated in both the ECRHS [21] and the LHS [6]. However, the criteria for recruitment of subjects and the methods of analysis were different between the ECRHS and the LHS. In our study of the LHS we used a nested case control design that allowed comparison of phenotypic extremes within a cohort of smokers selected for evidence of mild/moderate airflow obstruction. Therefore, disease severity genes were investigated in the LHS, while disease susceptibility genes were investigated in the ECRHS. This may be the basis of the discrepant results between the two studies [6,21]. Alternatively, our study may be a false negative result due to lower sample size. The previous studies that demonstrated an association of the (GT)_n _polymorphism and lung function decline had sample sizes of 749 [21], 101 [22], and 1390 [23] and therefore a larger sample size than that used in our study may be required to show the effect of this repeat polymorphism.

The tagSNP that was nominally associated with lung function decline in this study (rs2071749) was in perfect LD (r^2 ^= 1) with a SNP that was located in the promoter region of the *HMOX1 *gene (rs3761439). The promoter SNP was predicted to change the sequence of an NF-κB binding site and therefore may be the causal SNP for the association. We hypothesized that the G allele of rs3761439 was associated with relative resistance to lung function decline in smokers because it caused higher expression of the *HMOX1 *gene in response to specific stimuli.

Therefore, we conducted a functional analysis of the *HMOX1 *promoter. Two polymorphisms, rs2071746 (designated A-413T by Ono *et al. *[12]) and the (GT)_n _polymorphism, both of which were reported as functional [12,13], are located between rs3761439 and the transcriptional start site of the gene. Previously Yamada *et al. *showed in A549 cells that H_2_O_2 _exposure up-regulated the transcriptional activity of the *HMOX1 *promoter with S alleles of (GT)_n _but not with M or L alleles [13]. In lymphoblastoid cell lines, *HMOX1 *mRNA expression, HMOX1 activity, and resistance to oxidant-induced apoptosis were significantly higher in cells with the S/S genotypes than those with L/L [24]. However, it is still unknown whether the higher activities of HMOX1 were due to the (GT)_n _polymorphism, because the polymorphisms in this region are in strong LD with each other. Indeed, Ono *et al. *demonstrated that the A allele of rs2071746, but not the S allele of (GT)_n_, was associated with higher basal promoter activity in a reporter gene assay using bovine aortic endothelial cells [12].

To elucidate the functional consequences of promoter polymorphisms on *HMOX1 *inducibility by oxidative stress, we made nine plasmid constructs for reporter gene assays, based on the haplotype structure of the (GT)_n_, rs2071746, and rs3761439 polymorphisms. We used A549 cells and stimulated them with H_2_O_2 _or hemin. Hemin is known as an inducer of HMOX1, and generates reactive oxygen species. However, we did not detect any significant difference of the promoter activity among the nine plasmids in the transient transfection assays, in contrast to the previous *in vitro *functional studies [12,13,24]. Our negative results in the transient transfection assays may be related to regulatory sequences that were not included in the plasmids in this study but were included in the constructs utilized in the previous studies. We did not include a pGL3-basic vector in the experimental design as we were interested in the fold increase in gene expression i.e. comparing before and after H_2_O_2 _and hemin stimulation. This decision was motivated by the previous study by Yamada *et al. *[13] that demonstrated that the up-regulation of a reporter gene in response to H_2_O_2 _differed between promoters of different haplotypes. Nevertheless, this is a limitation of our study design.

## Conclusions

We found that none of the five tag SNPs in the *HMOX1 *region was related to lung function decline in smokers. This supports our previous results in the LHS where we investigated the (GT)_n _polymorphism. In addition, we did not demonstrate that the three investigated *HMOX1 *polymorphisms had an effect on the promoter activity of the gene in transient transfection assays and the rs2071749 SNP was not associated with *HMOX1 *in alveolar macrophages. These results suggest that the causal variant(s) that underlie the previous associations with COPD remain to be determined.

## Competing interests

The authors declare that they have no competing interests.

## Authors' contributions

GT carried out the reporter gene assays, statistical analysis and drafted the manuscript, FA performed the quantitative PCR, LA and JQH performed the SNP selection and genotyping assays, KS performed the statistical analysis of the genotypic data, JEC and NRA conceived of the Lung Health Study and helped draft the manuscript, RTA designed the alveolar macrophage study and helped to draft the manuscript, PDP and AJS conceived of the study and helped to draft the manuscript. All authors read and approved the final manuscript.

## Pre-publication history

The pre-publication history for this paper can be accessed here:

http://www.biomedcentral.com/1471-2350/12/117/prepub
